# Hand measurement data from human babies at birth, from 26 to 41 weeks estimated gestational age

**DOI:** 10.1016/j.dib.2016.03.089

**Published:** 2016-04-01

**Authors:** Emmanuelle Honoré, Thameur Rakza, Philippe Deruelle

**Affiliations:** a McDonald Institute for Archaeological Research, Downing Street Cambridge, Cambridge CB2 3ER, United Kingdom; b St. John׳s College, Cambridge CB2 1TP, United Kingdom; c Environnement Périnatal et Croissance EA 4489, Faculté de Médecine Henri Warembourg, Université Lille 2, Pôle Recherche, 59000 Lille, France; d Equipe Ethnologie Préhistorique, ArScAn – UMR 7041, Centre National de la Recherche Scientifique, Maison Archéologie et Ethnologie, 92 000 Nanterre, France

**Keywords:** Hand, Morphology, Measurements, Neonatalogy, Premature babies

## Abstract

This article displays measurement data from the hands of human babies, taken at birth. Measurements were made on 25 individuals born pre-term, from 26 to 36 weeks EGA (Estimated Gestational Age), and on 36 individuals born at term, from 37 to 41 weeks EGA. Data were collected in the Neonatal Unit of the CHRU Jeanne de Flandre (University Hospital) in Lille, France, between January and May 2014. Seven kinds of measures were taken with a medical caliper on the hand, palm and digits.

## **Specifications table**

**Table:** 

Subject area	*Biology*
More specific subject area	*Biometry*
Type of data	*Table*
How data was acquired	*Medical caliper*
Data format	*Raw*
Experimental factors	*Measurements were taken directly on hands with a medical caliper.*
Experimental features	*Measurements were taken in the first week of life.*
Data source location	*Neonatalogy Unit, CHRU Jeanne de Flandre (University Hospital), Lille, France*
Data accessibility	*Data is with this article*

## **Value of the data**

• Measurements taken at different gestational ages contribute to the elaboration of growth chart standards, useful for the assessment of dysmorphology or impaired development [1], [5].
• This data can be used as a reference series for comparative anatomy among juvenile primates.
• The variety of measures taken can make this data set suitable to be included in a study of functional morphology or biomechanics.
• Benchmark measures in hand morphometry are potentially useful for industrial applications, contributing to the improvement of gripping performances of pre-term babies items.

## 1 Data

Two series of measurements taken at birth on the hands of human newborns are displayed: a series from babies born pre-term, from 26 to 36 weeks EGA (Estimated Gestational Age), and a series from babies born at term, from 37 to 41 weeks EGA. Data was collected in the Neonatal Unit of the CHRU Jeanne de Flandre (University Hospital) in Lille, France, from January until May 2014. Seven measurement criteria were selected, concerning either lengths, widths or ray of the hand, the palm and the digits. They are recorded with the EGA, the sex and the weight of the individuals, regardless of the side – right hand or left hand (Tables 1 and 2).

**Table 1:** Measurement series on the hand of 25 human babies born pre-term (26–36 EGA), in mm.

**NAME**	**Surname**	**Estimated gestational age**	**Weight**	**Sex**	***W***_**i**_	***W***_**t**_	***R***_**t**_	***L***_**m**_	***L***_**p**_	***L***_**h**_	***W***_**h**_
RAH	OUN	34	1580	M	6	7.67	24.97	23.39	27.85	51.24	26.93
RAH	KAI	34	2125	M	7.85	7.64	32.01	25.13	31.64	56.77	28.73
KER	YOU	31	1100	M	5.82	6.23	25.8	20.96	29.04	50	23.57
GIL	THO	34	2005	M	6.92	8.2	35.35	23.27	31.3	54.57	27.48
DUF	THE	36	1380	M	6.58	7.39	30.67	22.96	27.82	50.78	27.7
EL	AMI	29	1410	M	5.89	6.66	18.75	22.9	20.34	43.27	26.42
BUD	ANI	30	1010	F	5.07	5.75	23.07	18.1	20.66	38.76	20.41
EL	YUS	35	1920	M	6.81	7.72	28.35	23.93	30.95	54.88	29.97
LEC	ALI	35	1200	F	7.29	7.52	25.28	22.17	26.11	48.28	25.15
MAN	ANG	33	1500	M	6.04	7.11	28.89	22.75	29.84	52.59	25.2
RAI	FOU	31	1300	M	5.89	6.72	17.34	22.82	33.34	45.16	23.89
GOU	LOU	26	850	M	4.04	4.65	27.56	21.36	20.07	41.43	21.4
SEN	ISA	29	900	M	5.04	5.7	27.25	19.7	24.47	44.17	20.55
LEG	EME	28	1060	F	6.37	7.01	22.39	20.58	24.49	45.07	24.18
GOU	LOU	29	1000	M	6.19	6.93	27.11	20.41	23.63	44.08	20.65
LAB	MAR	31	1640	M	7.94	8.13	29.24	26.35	22.21	48.56	24.45
LAB	SUS	31	1650	F	6.65	7.37	29.18	26.2	21.74	47.94	25.12
BRO	GEO	29	1600	M	8.33	8.37	23.97	22.44	25.4	47.84	25.83
FAR	ADR	31	1650	F	8.91	8.12	27.87	24.46	25.41	49.21	26.08
GUS	OCT	35	2160	F	6.23	7.95	34.9	25.25	30.14	55.39	28.81
GUS	ANA	35	2400	M	6.73	8	36.55	25.83	35.4	61.23	31.85
DEF	SAR	35	2090	F	6.53	7.3	34.02	25.95	28.67	54.62	28.2
DUT	MAT	36	2880	M	8.43	9.75	38.77	28.03	30.41	58.44	30.08
DEL	ELO	36	2780	M	7.75	8.67	38.57	27.39	29.87	57.26	33.26
CRE	STA	35	2630	M	7.17	8.68	32.7	26.77	31.66	58.43	29.81

**Table 2:** Measurement series on the hand of 36 human babies born at term (37–41 EGA), in mm.

**NAME**	**Surname**	**Estimated gestational age**	**Weight**	**Sex**	***W***_**i**_	***W***_**t**_	***R***_**t**_	***L***_**m**_	***L***_**p**_	***L***_**h**_	***W***_**h**_
BRA	CAM	37	3160	F	8.1	9.12	37.43	29.43	33.38	62.81	33.73
DEP	LUC	37	2250	M	7.38	8.06	30.94	25.96	31.47	57.43	30.83
TRO	NOL	38	2430	M	7.49	8.34	33.54	25.2	28.63	53.83	31.28
DAR	LIL	40	3150	F	7.52	8.16	35.32	26.89	29.66	56.55	31.98
BAL	LUC	41	3560	M	8.67	10.41	36.27	28.85	35.5	64.35	36.31
NIC	MAR	38	2470	M	8.12	9.9	36.32	27.78	30.21	57.99	31.08
MAI	FLA	40	3700	M	8.78	9.46	33.88	27.02	36.64	63.66	34.95
HEL	AIM	39	3160	F	8.53	8.64	34.08	26.13	37.87	64	32.68
LAN	BAP	37	2750	M	7.67	8.37	35.75	27.41	30.86	58.27	33.11
DAR	THO	39	3120	M	9.74	9.96	34.43	27.04	38.09	65.13	35.24
FON	TOM	39	4280	M	8.29	9.46	42	30.82	37.39	68.21	37.62
PAC	GUE	40	3160	M	8.64	9.63	38.06	27.53	36.08	63.61	33.33
CAE	ALE	39	3820	M	8.35	9.27	37.06	27.57	35.19	62.76	34
BOI	OLI	39	3560	F	8.34	9.62	36.94	28.38	32.27	60.65	34
VAC	LOU	39	3160	M	10.09	9.29	34.35	29.09	33.62	62.71	33.41
HAJ	WAL	41	3610	M	8.1	7.36	38.91	28.54	36.05	64.59	35.02
POU	BAP	41	3560	M	8.4	9.57	37.54	29.04	34.96	64	35.53
EL I	INA	40	3410	F	7.77	9.49	35.4	30.22	35.18	65.4	33.03
FOU	MAR	38	3700	M	9.55	9.59	38.38	31.42	36.54	67.96	34.96
ADD	HOU	40	3720	M	9.5	9.67	38.12	31.82	27.23	59.05	35.62
CHA	NOH	40	3730	F	8.26	9.18	42.43	28.1	36.05	64.15	34.5
PEC	BAK	40	4550	M	8.28	9.62	45.14	31.09	38.4	69.49	39.47
VAD	ANA	40	3490	F	7.26	8.75	38.03	27.24	35.42	62.66	35.16
HOU	AUD	39	3120	F	7.74	10.16	36.57	29.31	33.21	62.53	37.41
BEN	SER	41	2900	F	7.79	7.21	37.3	26.4	32.14	58.54	29.31
MUL	CEL	40	3990	F	8.11	9.49	40.48	30.57	35.49	66.06	36.33
CAT	THI	41	3890	M	8.06	10.08	43.55	31.42	36.46	67.88	36.4
EST	ILO	39	3060	F	8.14	8.32	36.07	28.18	35.46	63.64	32.67
EL	MAI	39	3340	F	9.36	9.5	36.94	27.5	35.51	63.01	35.28
SCH	KEN	39	3260	M	10.03	10.4	36.03	27.46	36.65	64.11	36.47
LE	EMI	40	3430	F	7.87	9.39	36.03	27.46	36.65	64.11	32.87
SAV	LOU	39	3220	M	7.24	8.03	40.7	29.81	37.53	67.34	33.81
BEN	IME	40	3450	M	8.93	8.87	34.06	30.56	36.55	64.66	34.25
SIB	MAO	37	1580	F	6.36	6.89	28.14	20	29	49	25.12
SIB	HOR	37	1635	M	5.96	7.2	27.25	20.64	23.14	43.78	25.06
CAL	NOE	38	2310	M	6.85	8.13	32.97	25.43	33.05	58.48	30.87

## 2 Experimental design, materials and methods

Seven measurements were taken on one hand of each individual with a medical caliper ([2], [3], [4]: Figure 3):

1. *W*_i_=width of the second digit (index) measured at the mid phalanx, just above the proximal inter-phalangeal joint.
2. *W*_t_=width of the first digit (thumb) measured at the middle of the proximal phalanx.
3. *R*_t_=Ray of the first digit (thumb) measured from the proximal end of the hand palm to the distal end of the thumb.
4. *L*_m_=length of the middle digit, measured from the base of the digit.
5. *L*_p_=length of the palm of the hand, measured from the proximal end of the hand to the distal end of the middle finger.
6. *L*_h_=maximal length of the hand, measured from the proximal end of the hand to the distal end of the middle digit.
7. *W*_h_=width of the hand, measured on the palm, just below the joint between the metacarpals and the proximal phalanges.

Measurements were made on the palmar face of the hand, in supination.
